# Cholangioscopy-assisted extraction of incarcerated abdominal drainage tube: a novel endoscopic approach

**DOI:** 10.1055/a-2599-7069

**Published:** 2025-06-03

**Authors:** Lechang Zhang, Yuemin Feng, Yong Chen, Maofeng Sun, Changqin Xu, Hongwei Xu, Shulei Zhao

**Affiliations:** 1 34708 Department of Gastroenterology, Shandong Provincial Hospital Affiliated to Shandong First Medical University, Jinan, China

**Fig. 1:**
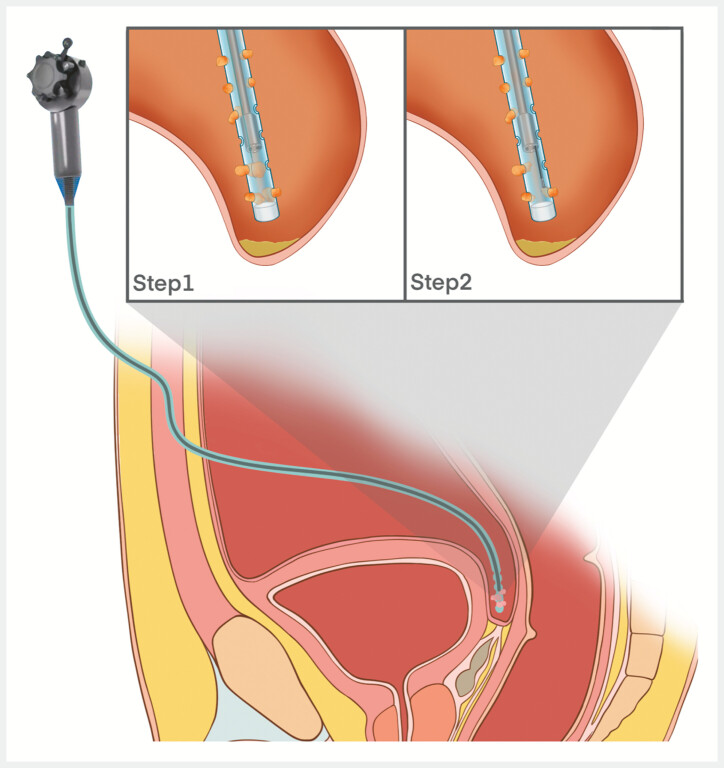
Cholangioscopy-assisted drainage tube extraction: procedural schematic.

A 56-year-old man diagnosed with adenocarcinoma of the ascending colon underwent laparoscopic right hemicolectomy, with intraoperative placement of a 24-Fr drainage tube (5-mm internal diameter) in the rectovesical pouch. On postoperative day 20, conventional approaches to extracting the drainage tube failed, despite radiographic confirmation of proper intraperitoneal positioning within the rectovesical pouch. This report describes how cholangioscopy-assisted intervention achieved successful extraction of the drainage tube ( Fig. 1 , Video 1 ).

Cholangioscopy-assisted extraction of incarcerated abdominal drainage tube.Video 1

**Fig. 2:**
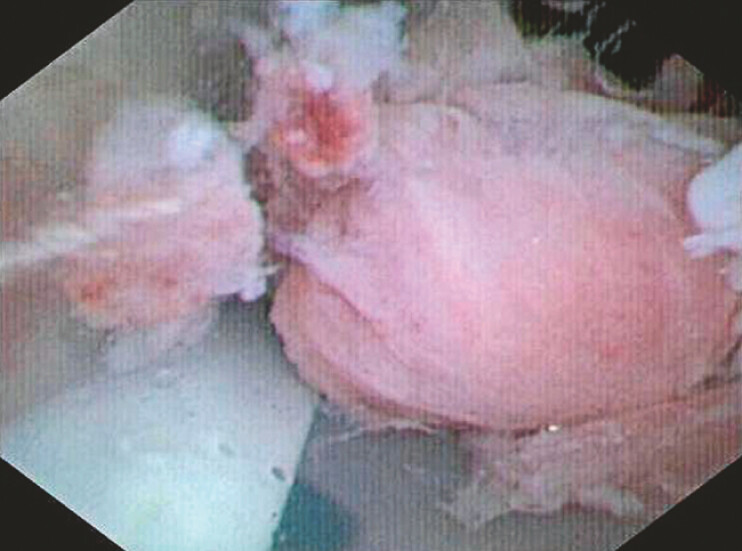
Ingrowth of fibroinflammatory tissue through drainage tube side ports with partial luminal obstruction.

**Fig. 3:**
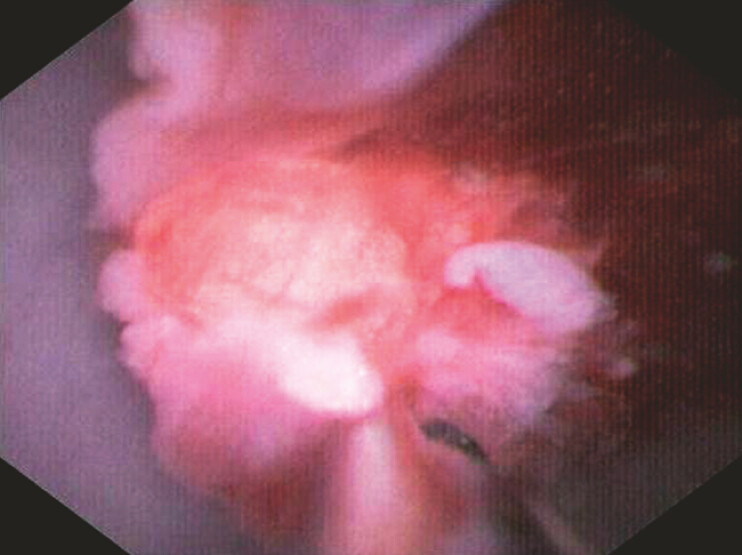
Debridement of fibroinflammatory ingrowth using miniature biopsy forceps.

**Fig. 4:**
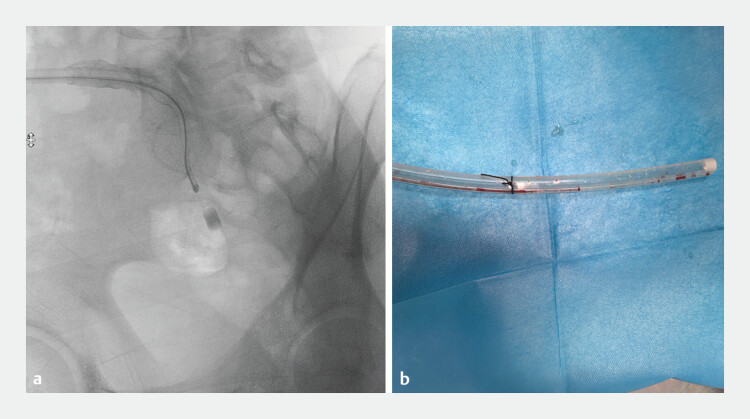
Tube extraction. **a** Fluoroscopy-guided drainage tube extraction with the cholangioscope tip reaching the tube end. **b** Retrieved drainage tube.

**Fig. 5:**
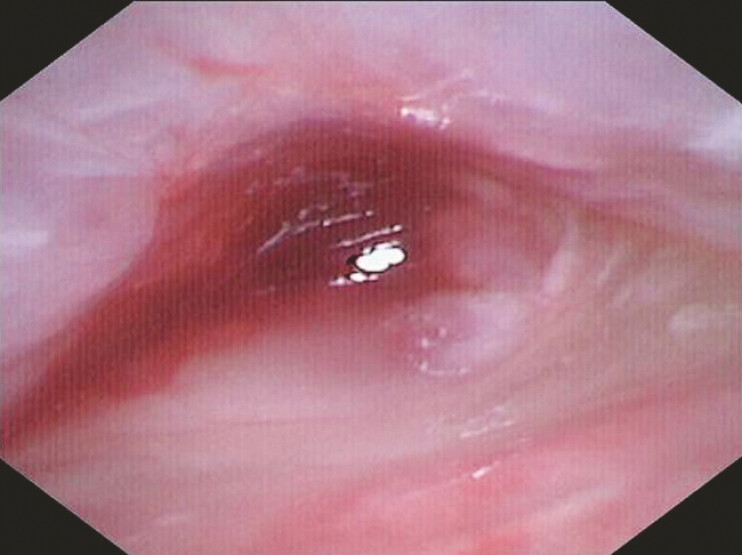
Post-extraction evaluation of the sinus tract via cholangioscopy, confirming intact architecture without residual debris or active hemorrhage.

Endoscopic evaluation using a 5-mm ultrathin gastroscope proved technically limited due to caliber mismatch, prompting utilization of a 9-Fr cholangioscope (eyeMAX, 9-Fr; Micro-Tech, Nanjing, China) for intraluminal inspection. The procedure revealed fibroinflammatory tissue ingrowth through side ports, forming a nail-shaped anchoring structure, establishing the pathological basis for tube retention ( Fig. 2 ). Under direct visualization through the cholangioscope, a staged debridement protocol was systematically performed: sequential extraction of obstructing fibrotic tissues was accomplished through consecutive applications of snares followed by grasping forceps via the drainage lumen, and meticulous dissection of side-port adhesions using miniature biopsy forceps through the endoscope working channel, with samples sent for histopathological analysis ( Fig. 3 ). Following clearance of approximately 80% of the tissue, the cholangioscope tip maintained close apposition to the drainage tube terminus during controlled withdrawal to preserve sinus tract integrity, culminating in successful tube extraction ( Fig. 4 ). Post-procedural assessment through the established tract confirmed intact sinus architecture without residual debris or active hemorrhage, while cutaneous examination revealed no iatrogenic laceration ( Fig. 5 ).

This case highlights critical technical innovations including the lumen-to-sinus visualization paradigm preventing blind manipulation-induced tissue trauma, phased debridement minimizing shear stress on compromised tube walls, and endoscopic velocity-controlled extraction optimizing safety through continuous anatomical feedback. This represents the first documented application of cholangioscopy-assisted drainage tube extraction in surgery complications, providing a novel paradigm for managing similar iatrogenic incarceration scenarios.

Endoscopy_UCTN_Code_TTT_1AQ_2AF

